# Electrochemical Performance of Graphene Oxide/Black Arsenic Phosphorus/Carbon Nanotubes as Anode Material for LIBs

**DOI:** 10.3390/ma15134576

**Published:** 2022-06-29

**Authors:** Yanyan Hou, Shufang Ma, Yang Xu, Shuai Zhang, Xiaodong Hao, Bingshe Xu

**Affiliations:** 1 Materials Institute of Atomic and Molecular Science, Shaanxi University of Science and Technology, Xi’an 710021, China; bs1802005@sust.edu.cn (Y.H.); m18209180923@163.com (Y.X.); zhangshuai20210830@163.com (S.Z.); hao.xiaodong@sust.edu.cn (X.H.); xubingshe@sust.edu.cn (B.X.); 2 Key Laboratory of Interface Science and Engineering in Advanced Materials of Ministry of Education, Taiyuan University of Technology, Taiyuan 030024, China

**Keywords:** black arsenic phosphorus, 2D materials, carbon nanotubes, lithium-ion batteries

## Abstract

As a new two-dimensional material, black arsenic phosphorus (B-AsP) has emerged as a promising electrode for lithium-ion batteries (LIBs) due to its large theoretical capacity and ability to absorb large amounts of Li atoms. However, the poor electronic conductivity and large volume expansion during the lithiation/delithiation process have largely impeded the development of B-AsP electrodes. In this study, graphene oxide (GO)/B-AsP/carbon nanotubes (CNTs) with remarkable lithium-storage property were fabricated via CVD and ultrasound-assisted method. The electrochemical behavior of the GO/B-AsP/CNTs was investigated as an anode in lithium-ion batteries. From the results, as a new-type anode for LIBs, GO/B-AsP/CNTs composite demonstrated a stable capacity of 1286 and 339 mA h g^−1^ at the current density of 0.1 and 1 A g^−1^, respectively. The capacity of GO/B-AsP/CNTs was 693 mA h g^−1^ after 50 cycles, resulting in capacity retention of almost 86%. In addition, the stable P-C and As-C bonds were formed between B-AsP, GO, and CNTs. Thus, volume expansion of B-AsP was alleviated and the capacity was increased due to the confining effect of GO and CNTs.

## 1. Introduction

Lithium-ion batteries (LIBs) with high performance have attracted considerable attention over the past decades to meet the demands of laptop computers, portable devices, and electric vehicles [1,2,3,4]. Currently, as a commercial LIB anode material, graphite has shown an electrochemical performance that is relatively stable. However, graphite has a theoretical capacity of only 372 mA h g^−1^ [5], making it difficult to meet the huge demand for future energy storage markets. The theoretical capacity of other anode materials, such as transition metal oxides [6,7], silicon matrix composites [8,9,10,11], metal nitrides [12,13], and Sn-based oxides [14,15,16] is relatively high (Table 1), but these suffer from excessive volume expansion and low conductivity. Therefore, there still is an urgency to develop new high-capacity anode materials for LIBs [17].

Graphene oxide (GO) is an intermediate product between graphene and graphite, with a quasi-two-dimensional layered structure [18], and its surface contains abundant functional groups such as hydroxyl, epoxy, carboxyl, and carbonyl [19]. These functional groups give GO good dispersion properties, while also making it a suitable supporting material, offering not only abundant binding sites for other substances to recombine, but also preventing the aggregation of nanoparticles and causing a wrinkled appearance. On one hand, the volume expansion problem can be inhibited, and accommodate the huge anisotropic changes during charging/discharging processes; on the other hand, the conductivity of materials is increased and subsequently improves the electrochemical performance.

The pnictogens (P, As, Sb, and Bi) have garnered significant attention due to high theoretical capacities based on the formation of Li_3_X (X = P, As, Sb, or Bi). P, Sb, and even Bi have been studied as anode materials for reversible Li or Na cells [20,21,22,23,24,25,26,27]; however, arsenic has largely been ignored. The alloying of Li with arsenic was first demonstrated by Besenhard et al. in 1975 [28]. Only recently, years after Besenhard’s initial report, has another study appeared on elemental arsenic-based Li-ion anodes [29]. The absence of arsenic anode research may be presumably due to environmental concerns. However, each year thousands of metric tons of arsenic will be industrially used in wood preservatives, agricultural chemicals, lead acid batteries, and semiconductors [30]. Though arsenic is toxic, the maximum contaminant level in drinking water is 10 μg L^−1^, nearly the same as Pb (15 μg L^−1^) [31], which is present in large quantities in ubiquitous Pb-acid motor vehicle batteries. As with lead–acid batteries, effective recycling can make the use of arsenic-based batteries environmentally acceptable.

In recent years, with the rapid development of layered materials, two-dimensional materials have attracted increasing attention [32,33,34]. Black arsenic phosphorus (B-AsP) has attracted significant due to its unique characteristics and flexible two-dimensional structure. The terminal compounds formed in the lithium state of B-AsP consist of Li_3_P and Li_3_As, which have shown high theoretical specific capacities (2596 mA h g^−1^, 2052 mA h g^−1^) and a low diffusion barrier (0.08 eV). Nanostructured materials have the advantages of large surface area, high diffusion rate, and stable cycling behavior, which can improve the electrochemical performance of LIBs. In the previous work, the electrochemical performance of B-AsP in a lithium-ion battery was studied. Due to the volume expansion of B-AsP [27,35,36,37,38], the cycling performance and rate performance of B-AsP electrode were poor. Therefore, to reduce the volume expansion of B-AsP, B-AsP crystals were stripped into single or multi-layers. Because lithium ions are stored between layers during charge-discharge, layer reduction could alleviate the volume changes caused by intercalation, protecting the structure of the electrode material. In this work, on the basis of B-AsP, the GO/B-AsP/CNTs composite was prepared by ball milling. GO offered excellent characteristics and rich functional groups, thus, during the process of ball milling, P-C and As-C bonds formed between B-AsP and GO. The P-C and As-C bonds strengthens the connection between the B-AsP and carbon. The P-C and As-C bonds played a role in stabilizing the structure of B-AsP, as the anode of LIBs, for studying the electrochemical performance of the GO/B-AsP/CNTs composite material. In addition, we determined role of the P-C and As-C bonds and electrochemical reaction mechanism between Li-P and Li-As.

**Table 1:** Energy storage properties of alloy-based anodic materials for lithium-ion batteries.

	Theo. Spec. Cap (mAh g^−1^)	Theo. Vol. Cap. (mAh g^−1^) [39]	Av. Charge Potential (V vs. Li^+^/Li)	Av. Discharge Potential (V vs. Li^+^/Li)	Vol. Variation (%)	Lithiated Phase
Graphite	372	837	0.1–0.1	0.17	12	LiC_6_
Si [40]	4200	9786	0.2	0.45	420	Li_4_._4_Si
Ge [41]	1600	8645	0.4	0.65	370	Li_4_._4_Ge
Sn [15]	994	7216	0.4	0.6	257	Li_4_._4_Sn
BP [37]	2596	2266	0.45	0.9	300	Li_3_BP
As [36]	1073	2057	0.9	1.1	182	Li_3_As
Sb [42]	660	1750	0.8	1	150	Li_3_Sb
Bi [43]	386	3765	0.7	0.9	215	Li_3_Bi

## 2. Materials and Methods

### 2.1. Materials Preparation

The B-AsP materials were synthesized via mineralization-assisted gas phase transport. The raw materials were a mixture of gray arsenic (99.9999%) and red phosphorus (99.999%), and a mixture of SnI_4_ (99.999%) and Sn (99.999%) was used as a mineralizer. All chemicals were enclosed in an evacuated silica glass quartz tube (length: 100 mm; internal diameter: 85 mm). The gray arsenic and red phosphorus with molar ratios of 83:17 was marked as B-As_0.83_P_0.17_. The materials were heated to 750 °C within 8 h and held for 20 h; they were then cooled to 550 °C within 2 h and held for 1 h. The samples were cooled to room temperature naturally within 5 h. The B-AsP crystals could be found at the cold end of the silica glass tubes. The methods were according to the methods described in previous work [44].

For the GO/B-AsP/CNTs composite materials: First, the B-AsP and GO were ground and ultrasonicated for 2 h, with an ultrasonic power value of 100 W. The dispersion of the GO/B-AsP composite material was centrifuged in a centrifuge at 1509 rcf for 10 min. The supernatant was collected, and freeze-dried to obtain the powders of GO/B-AsP. The GO/B-AsP powder and the CNTs were placed in a spherical ink tank and ball milled at 500 rpm min^−1^ for 6 h, and then the mixture was dried under a vacuum for 24 h. The black powder consisted of the GO/B-AsP/CNTs composite.

### 2.2. Material Characterization

To evaluate the crystallinity of the B-AsP, CNTs, GO, and GO/B-AsP/CNTs, the powder X-ray diffraction (XRD) patterns were measured at an angular range of 2θ from 10° to 90° by a Rigaku D/MAX-2500/pc with a Cu–Kα (λ = 1.5418Å) line at a tube voltage of 40 kV and a tube current of 40 mA. To analyze the molecular structure of the sample, the Raman spectra (Raman) were measured at a scanning range of from 100 to 5000 cm^−1^ by the laser wavelength of 525 nm and the laser power of 5 W. Surface elemental analysis was performed by X-ray photoelectron spectroscopy (XPS, Tokyo, Japan, PHI Quantera SXM-CI, Ulvac-Phi) using a monochromatized Al–Kα line with an emission current of 2.7 mA and an acceleration voltage of 15 kV. The morphology and microstructure of the samples were characterized by a field-emission scanning electron microscope (SEM, Eschborn, Germany, FEI Verios 460) and transmission electron microscope (TEM). One drop (10 µL) of a redispersed sample was directly deposited onto a carbon film (~5 nm). Low-magnification TEM images were taken using JEM-2100Plus (Tokyo, Japan, JEOL Co., Ltd.) with an accelerating voltage of 200 kV.

### 2.3. Electrochemical Tests

For electrode preparation, the active material (70 wt.%), acetylene black (20 wt.%), and polyvinylidene difluoride (PVDF) (10 wt.%) were mixed in the N-methyl pyrrolidinone (NMP) solvent and stirred evenly. The mixture was coated on copper foil and maintained at 60 °C in a vacuum for 24 h. The obtained electrode was stamped into small 14 mm diameter discs, and each piece contained approximately 2.3 mg of the active substance. Metal lithium foil was used as the counter electrode. The electrolyte was 1 mol/L LiPF_6_ in ethylene carbonate (EC), propylene carbonate (PC), and diethyl carbonate (DEC) with volume ratio of 1:1:1. The batteries were assembled in an argon-filled glove box.

We used the land CT 21001A system (Wuhan Jinnuo, China) to test the galvanostatic charge/discharge of battery and the test voltage window was 0.01-3 V. CHI660C electrochemical workstation was used to measure the cyclic voltammetry (CV) curves and electrochemical impedance spectra (EIS). The CV curves were tested at scanning rate of 0.1 mV s^−1^ between 0.01 and 3.0 V, and the EIS was tested in a frequency of 100 kHz-0.01 Hz. The samples were tested in three groups, one group of three repetitions. All the tests were carried out at room temperature (25 °C).

## 3. Results and Discussion

**Figure 1:**
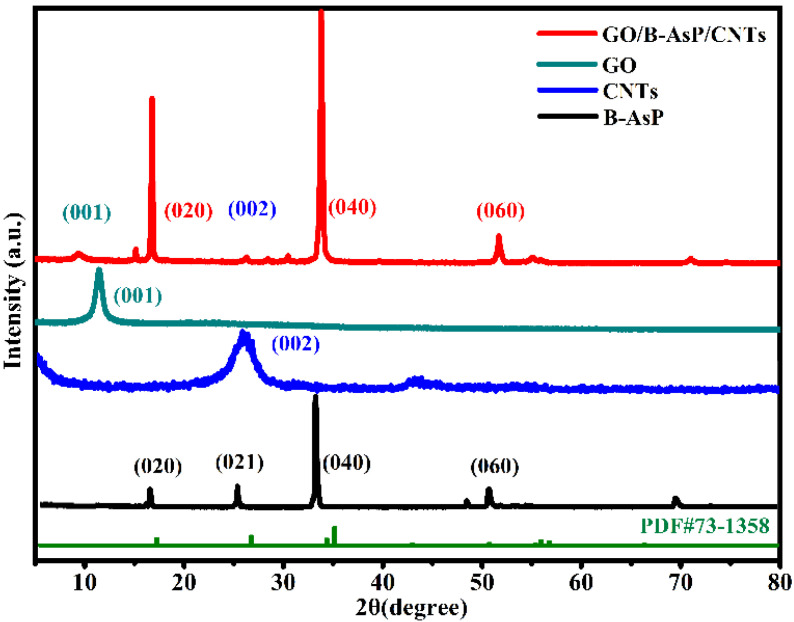
XRD patterns of B-AsP, CNTs, GO, and GO/B-AsP/CNTs.

The XRD patterns of GO and GO/B-AsP/CNTs were studied and are shown in Figure 1. GO had a strong characteristic peak at 10°, corresponding to the GO (001) crystallographic planes. There were no other impurity peaks, indicating that GO had high purity. In the composites, the characteristic diffraction peak of GO shifts to a small angle. This indicates that the structure of GO has changed, and the layer spacing of GO increases during the combination process with B-AsP. For B-AsP, it showed four characteristic diffraction peaks of 16.5° (020), 26.5° (021), 33.5° (040), and 52.5° (060), and it had an obvious layered structure and good crystallinity. These correspond to the diffraction pattern of black phosphorus (BP) (PDF#73-1358) in the database. Notably, The B-AsP and BP have similar crystal structures. B-AsP is composed of phosphorus atoms and arsenic atoms, and the atomic radius of arsenic is larger than the phosphorus atomic. In the process of black arsenic-phosphorus synthesis, due to the change of lattice structure caused by the substitution of arsenic for phosphorus, the diffraction peaks of B-AsP become broad and shift toward lower angle [44]. In the composites, in addition to the characteristic peaks of B-AsP and GO, the characteristic peak of CNTs appeared at 26.4° (002). The pattern is in good agreement with CNTs with JCPDS No. 41-1487. The five peaks at 10° (001), 16.5° (020), 26.4° (002), 33.5° (040), and 52.5° (060) indicated the simultaneous presence of B-AsP, CNTs, and GO. The coexistence of B-AsP, GO, and CNTs in the composites suggested the successful preparation of the GO/B-AsP/CNTs composites.

**Figure 2:**
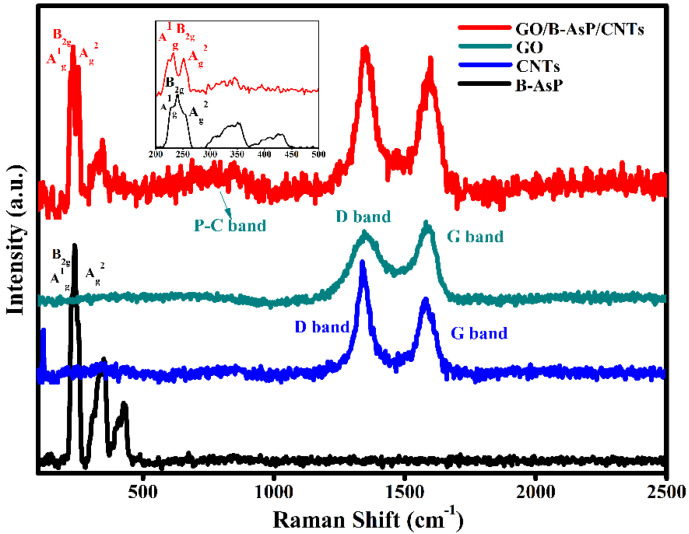
Raman patterns of B-AsP, CNTs, GO, and GO/B-AsP/CNTs.

Figure 2 shows the Raman spectra of the GO and GO/B-AsP/CNTs composite samples. There were three major peaks in the B-AsP spectra at 226, 242, and 256 cm^−1^, which corresponded to out-of-plane vibration modes of Ag^1^, and in-plane vibration modes B_2g_, and Ag^2^. These peaks were consistent with the previous report [44,45]. In the spectrum of GO/B-AsP/CNTs, in addition to the three major peaks of B-AsP, the D and G bands of GO were clearly observed at 1345 and 1582 cm^−1^, which was consistent with the Raman spectra of GO, as observed [46,47]. The D peak was stronger than the G peak, indicating that there were some oxygen-containing functional groups on GO. In the composite, the characteristic peaks of GO, B-AsP, and CNTs exist at the same time, which was consistent with the XRD analysis. At the same time, as presented in Figure 2, the characteristic peak of P-C bonds appeared at 600-900 cm^−1^. P-C bonds strengthened the connection between the B-AsP and carbon [48].

**Figure 3:**
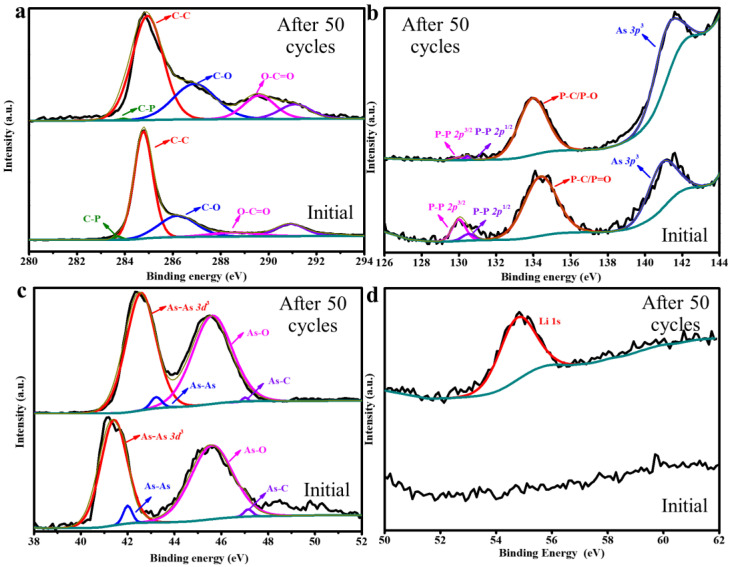
XPS spectra of GO/B-AsP/CNTs before and after charge-discharge cycles. (**a**) C 1s, (**b**) P 2p, (**c**) As 3d, and (**d**) Li 1s.

To further analyze the functional groups in the GO/B-AsP/CNTs composite, the XPS patterns of the GO/B-AsP/CNTs composite were measured as shown in Figure 3. As indicated in Figure 3a, in the C 1s spectra, the peak area located at binding energies of 283.5, 284.5, 286.1, 288.1, and 289.9 eV corresponded to the C-P bonds, sp^2^/sp^3^ C-C, C-O bonds, and −O-C = O bonds, respectively [49]. The increased intensities corresponded to O-C = O groups. It is shown that the strength of oxygen and inner carbon atoms increases after charge–discharge cycling, and the increment in sp^3^ carbon atoms is due to the adhesion of oxygen. The peak that appears at 290.7 eV in the XPS C 1s spectrum appeared due to the equipment problem. In the XPS spectrum of 2p spectra (Figure 3b), we observed double peaks representing P2p_1/2_ and P2p_3/2_ at 130.6 and 130 eV, respectively, which corresponded to the P-P bonds in B-AsP [50]. However, the strong peak appeared on the initial anode at 134.2 eV, which was formed by the P=O bond in P_2_O_5_, possibly by exposure to air. Except for the P–P bonds, the peak at 134.2 eV corresponded to the P-C and P-O-C bonds. The peak generated by P = O bond shifted to a lower binding energy, corresponding to P-O bond, after charge-discharge cycling. In the XPS spectrum of As (Figure 3c), we observed double peaks representing As 3d at 41.8 eV and 42.1 eV, which corresponded to the As-As bonds in B-AsP. As shown in Figure 3c, after 50 cycles, the peak of As-As bond shifts to the low binding energy because the valence state of As changes and the As-As bond shrinks during the charging-discharging process. However, the peak appeared on the initial anode at 45.5 eV, which was formed by the As-O bond in arsenic oxide, may be formed by exposure to air. The As-O bond shifted to a lower binding energy after discharge. The change of the binding state of arsenic oxide is due to the reaction of Li ions and/or deposited Li metal with oxygen atoms on arsenic particles during the charging and discharging process, resulting in the change of the oxidation state of arsenic oxide, in which arsenic oxide may be between As_2_O_5_ (oxidation state; 5^+^) and As_2_O_3_ (oxidation state; 3^+^) [51]. The peak that appears at 47.1 eV corresponded to the As-C bonds. Thus, arsenic, phosphorus was potentially stably trapped in the inner wall of carbon nanotubes by the presence of oxygen functional groups. XPS spectra of Li 1s (Figure 3d) confirmed the peak of lithium metal at 54.3 eV. The formation of Li_3_P, LiP and Li_3_As, LiAs was not observed at Li 1s. Therefore, it suggests that reversible reactions occur between Li^+^ and GO/B-AsP/CNTs anode during charge–discharge cycling. The oxidation reaction of oxygen-containing functional groups on the surface of GO and B-AsP led to the emergence of C-O, P-O, and As-O bonds. The C-O, P-O, and As-O bonds increased the structural disorder of the composite and reduced the chemical reaction energy barrier, which further promoted the formation of P-C and As-C bonds. The P-C and As-C bonds can protect the B-AsP during the lithium intercalation-delithiation process, which suppressed the structural destruction resulting from volume expansion and ensured the structural stability of the electrode material.

**Figure 4:**
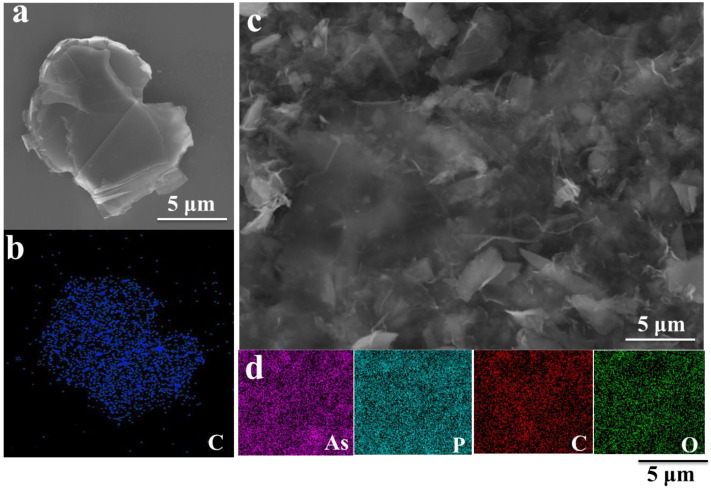
(**a**) SEM image and (**b**) EDS spectrum mapping of GO, (**c**) SEM image, and (**d**) EDS spectrum mapping of GO/B-AsP/CNTs.

Figure 4a,c show the SEM of GO, GO/B-AsP/CNTs composite. The energy dispersive spectroscopy (EDS) maps are in Figure 4b,d. As shown in Figure 4a, GO was composed of regular thin slices with clearly visible edges. Figure 4b is the EDS of GO. The morphology of GO/B-AsP/CNTs composites is shown in Figure 4c. The results revealed that the GO and CNTs combined with layered B-AsP, and the distribution of B-AsP, GO, and CNTs was also relatively uniform, without obvious agglomeration and accumulation. EDS mapping images showed the distribution of elements As, P, and C in the GO/B-AsP composites materials, as shown in Figure 4d. The P, As, C, and O elements were evenly distributed in GO/B-AsP without obvious agglomeration or accumulation. In addition, GO was coated and intercalated on the surface and inside of B-AsP, forming an interconnected structure and playing a supporting role. This suppressed structural destruction by volume expansion and ensured the structural stability of the electrode material.

**Figure 5:**
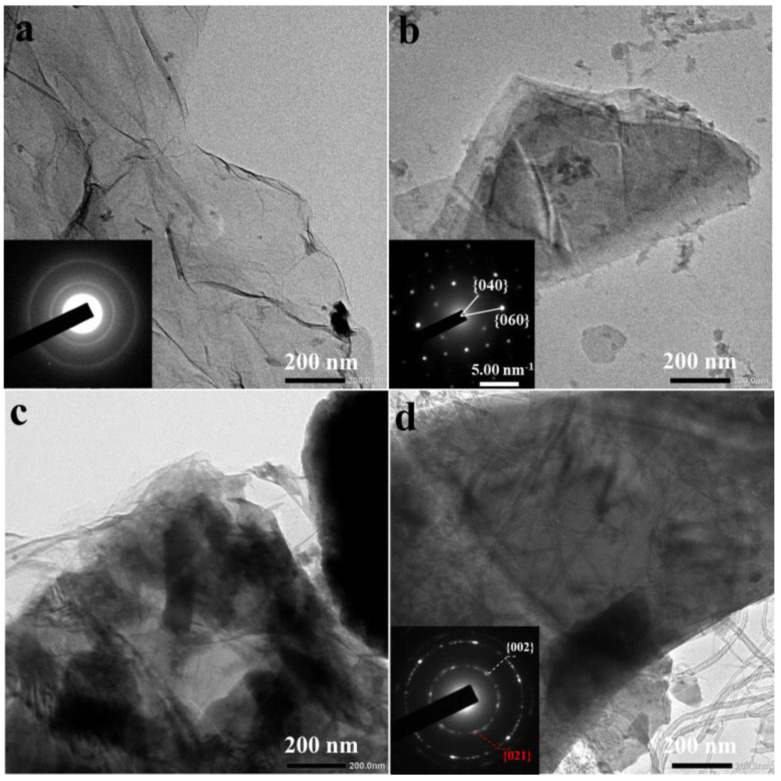
TEM of (**a**) GO, (**b**) B-AsP, (**c**) GO/B-AsP, and (**d**) GO/B-AsP/CNTs (inset: SAED pattern).

Figure 5 shows the TEM of GO, B-AsP, GO/B-AsP, and GO/B-AsP/CNTs composite. As can be seen from Figure 5a, GO presents thin paper folds without agglomeration, and the diameter is micron. In Figure 5b, the microstructure of the B-AsP material can be clearly seen. The B-AsP was composed of regular thin slices with clearly visible edges, and this structure has weaker van der Waals interactions between the layers [52]. Figure 5c shows the low-power TEM of GO/B-AsP. It can be seen that GO was covered or interspersed with B-AsP, which is consistent with the results of SEM analysis. At the same time, the composite material has the phenomenon of agglomeration. During the ball milling process, the material was repeatedly sheared, peeled off, and then reunited. As shown in Figure 5d, the carbon nanotubes were interspersed between layers of GO and B-AsP, which were covered by GO. The selected-area electron diffraction (SAED) image of B-AsP is shown in the inset of Figure 5b. It shows that the B-AsP has a layered structure, which is consistent with XRD. The inset in Figure 5d shows the {021} lattice plane of B-AsP, and the {002} lattice plane of carbon nanotubes, which illustrates the combination of B-AsP and carbon materials. This type of penetration and coating structure could alleviate the volume expansion of B-AsP during the charge-discharge process, ensuring the structural stability of the electrode material.

**Figure 6:**
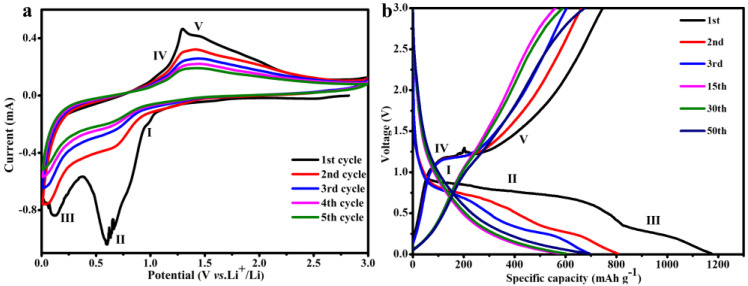
(**a**) The cyclic voltammograms of GO/B-AsP/CNTs at 0.2 mV s^−1^. (**b**) The galvanostatic charge-discharge curves of GO/B-AsP/CNTs.

The CV profiles presented in Figure 6a could be used to understand the lithium-storage mechanism of the GO/B-AsP/CNTs composites at a scanning speed of 0.2 mV s^−1^ and a voltage window of 0.01-3 V. As shown in Figure 6a, the oxidation and reduction peaks in the figure corresponded to the two processes of delithiation and intercalation in the electrode material. In the first curve, there were three obvious reduction peaks, where the first peak was near 1.05 V, but this disappeared in the subsequent scanning process. The peak represented the formation of the solid electrolyte interface (SEI) film during discharge. With further lithium intercalation, peak II appeared near 0.6 V, which consisted of the formation process of LiP, Li_2_P, LiAs, and Li_2_As [29,53]. As the lithium ions approached saturation, more lithium combined with phosphorus and arsenic to form Li_3_P and Li_3_As [53,54], which corresponded to peak III in the curve. During the charging process, the lithium ions were reduced and separated from the anode electrode material to reach peaks IV and V on the curve. At this time, the remaining lithium ions formed irreversible LixP and LixAs compounds with B-AsP, thus causing irreversible capacity. The trends of the CV curves were similar, starting with the second cycle, where the reversible lithiation and delithiation signature of AsP (AsP + xLi^+^ + xe^−^ → Li_x_AsP) [54] appeared consistently as a pair of peaks, with potentials around 0.7 and 1.1 V, respectively. 

The galvanostatic charge-discharge curves of GO/B-AsP/CNTs composites were tested to investigate the electrochemical properties of the new electrode. The five peaks in Figure 6a corresponded to the five charge-discharge plateaus in Figure 6b. After the first cycle of charge-discharge, the reversible capacity of the GO/B-AsP/CNTs composite was only 800 mAh g^−1^ in the second cycle, which was mainly in the formation of the SEI film. After 50 cycles, the reversible capacity was 693 mAh g^−1^, and the reversible capacity reached 86%. The results showed that after the activation of electrode and formation of stable SEI layer, the structures of GO/B-AsP/CNTs composites remained stable during subsequent cycles.

**Figure 7:**
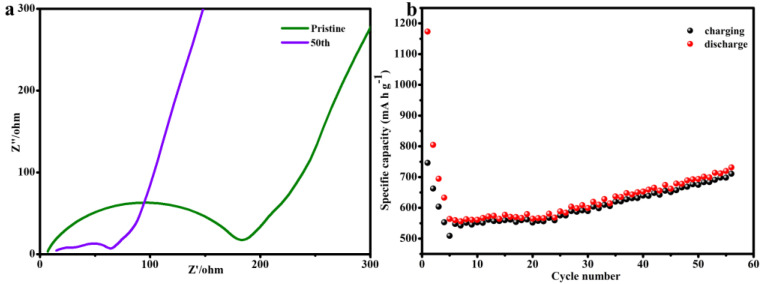
(**a**) Electrochemical impedance spectroscopy (amplitude: 5 mV, 100 kHz–0.01 Hz). (**b**) Cycle performance of GO/B-AsP/CNTs at 0.1 A g^−1^ for 50 cycles.

Figure 7a shows the EIS spectra of GO/B-AsP/CNTs electrodes after 50 cycles. The semicircle in the high-frequency region was ascribed to charge-transfer resistance (Rct) and the inclined line at the low-frequency region was ascribed to Warburg resistance. The Rct of GO/B-AsP/CNTs electrode is 180.6 Ω. After discharging to 3.0 V, double semicircles in the high-medium-frequency region were discovered. The first semicircle at a higher frequency and second semicircle were related to the formation of SEI (Rs) and Rct, respectively. The Rct of GO/B-AsP/CNTs electrode after discharging to 50 cycles (60.5 Ω) definitely decreased due to the electrode-electrolyte activation and lithium-ion low-diffusion resistance. Therefore, GO/B-AsP/CNTs composites had good electrical conductivity, which further improved the electrochemical performance. To confirm the cyclability of the GO/B-AsP/CNTs anode for LIBs, galvanostatic charge-discharge curves were recorded at 100 mA g^−1^ (Figure 7b). It can be seen from the figure that the first cycle charge–discharge capacity of the GO/B-AsP/CNTs composite is 1173 mA h g^−1^ and 746 mA h g^−1^. After 50 cycles, the capacity was 693 mA h g^−1^, and the electrode remained relatively stable. Thus, the electrochemical performance of GO/B-AsP/CNTs composite was better than the continuously degraded B-AsP electrode [44]. This was mainly the coating and protection of B-AsP by graphene oxide and carbon nanotubes, which formed P-C and As-C bonds, causing the structure of B-AsP to be more stable and ensuring the stability of electrode materials. Thus, more lithium ions reacted with arsenic and phosphorus atoms in B-AsP, increasing the reversible capacity. The results of Raman and XPS provide evidence for the formation of P-C and As-C bonds.

**Figure 8:**
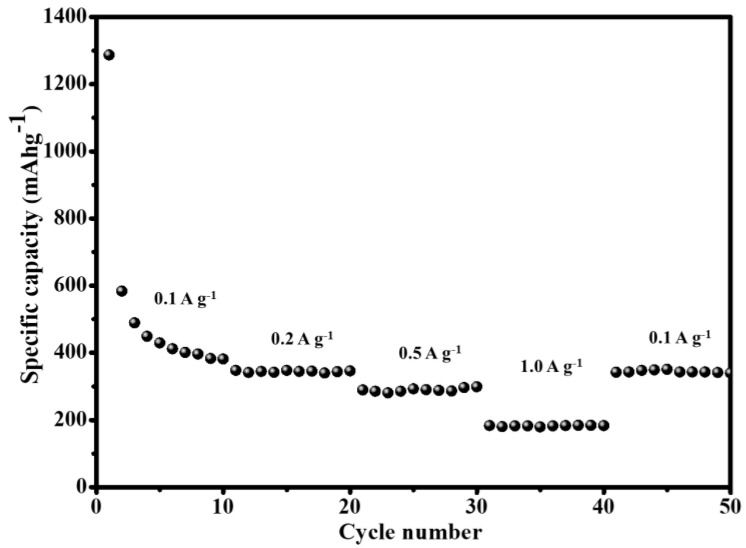
Rate performance at different current densities.

The GO/B-AsP/CNTs also exhibited a superior rate capability (Figure 8). The electrode material shows a high capacity of 1286, 381, 345, and 298 mA h g^−1^ at the current density values of 0.1, 0.2, 0.5, and 1 A g^−1^. The current was restored to 0.1 A g^−1^, and the capacity of the electrode material was restored to 339 mAh g^−1^. The results showed that the structure of electrode material was not damaged by the change of current. On the other hand, it can also be shown that with the formation of P-C and As-C bonds, the structure of the electrode material was more stable.

**Figure 9:**
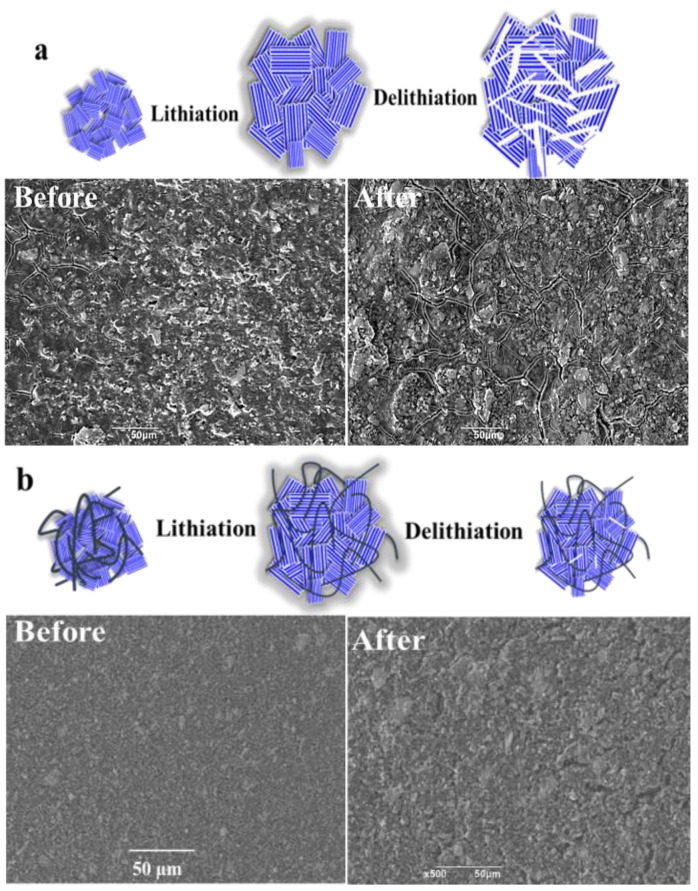
Schematic diagram of surface structure changes of lithiation and delithiation reaction and SEM images of B-AsP (**a**) and GO/B-AsP/CNTs (**b**) electrodes before and after 50 cycles.

Figure 9a,b show the surface structure changes after charging and discharging of B-AsP and GO/B-AsP/CNTs electrodes. Figure 9a shows the surface structure of the B-AsP electrode before and after 50 cycles of cycles. It can be seen that after 50 cycles, the surface of the B-AsP electrode was fractured, while the surface of the GO/B-AsP/CNTs still maintained good morphology and structure (Figure 9b). In general, B-AsP materials form Li_3_P or Li_3_As during the lithiation process, and the theoretical band gap of Li_3_P is 1.74 eV [53], which is much larger than the band gap of the pristine B-AsP (0.19 eV) [29], indicating that the electronic conductivity of B-AsP decreases after intercalation with lithium. B-AsP have huge volume changes during (de)intercalation and (de)alloying reactions in lithium-ion batteries [27,35]. The repeated expansion and contraction process leads to structural fracture and discontinuous contact of the B-AsP, which finds it impossible to recover to its original state. Thus, it not only destroys the capacitance performance, but also reduces the specific capacity of the B-AsP. After the introduction of GO and CNTs into B-AsP, the GO and CNTs play the role of support so that it can limit the volume expansion during the lithiation process and offset the volume decrease during the delithiation reaction. B-AsP was closely connected with the GO, CNTs, and the formation of P-C and As-C bonds, which endow the GO/B-AsP/CNTs with good structural stability and improve the cycling performance. Moreover, introduction of carbon-based materials will increase the electrical conductivity of the GO/B-AsP/CNTs owing to the high electrical conductivity of carbon-based materials, which helps to reduce the contact resistance of the material and contributes to the performance of the GO/B-AsP/CNTs.

## 4. Conclusions

In summary, we successfully prepared GO/B-AsP/CNTs composites, which were subsequently examined by XRD, Raman, XPS, SEM, and TEM techniques. The analysis results showed that the GO, CNTs, and B-AsP were composites, and that GO and CNTs were distributed in the layered B-AsP. The P-C and As-C bonds made the structure of B-AsP more stable. In the electrochemical performance test, the first cycle charge-discharge capacity of the GO/B-AsP/CNTs composite showed a high capacity of 1173 and 746 mAh g^−1^. After 50 cycles, the electrode material capacity was 693 mA h g^−1^, which remained relatively stable. In the action of GO and CNTs, the volume changes and structural strain of B-AsP were limited during the charge-discharge process, which provided basic support for the energy storage research of B-AsP-based composites in principle.

## Data Availability

The data presented in this study are available on request from the corresponding author.
